# Probing the microRNA landscape in cadmium chloride induced renal toxicity through an in silico approach

**DOI:** 10.1038/s41598-025-11473-1

**Published:** 2025-07-12

**Authors:** Arnab Mukherjee, Sai Eashan Vankamamidi, Mukunthan KS

**Affiliations:** Department of Biotechnology, Manipal Institute of Technology, Manipal Academy of Higher Education, Manipal, Manipal India

**Keywords:** Cadmium chloride, Renal toxicity, miRNA, Argonaute, Molecular dynamics simulation, RNA, Structural biology, Biochemical reaction networks, Gene regulatory networks, Microarrays, Network topology

## Abstract

Cadmium chloride (CdCl₂), a highly toxic environmental pollutant, significantly impacts kidney health, particularly in the proximal tubular cells, where it induces oxidative stress and lipid peroxidation. The specific mechanisms underlying cadmium toxicity remain unclear. It is hypothesized that it is mediated by microRNAs (miRNAs). These non-coding RNAs regulate gene expression by promoting mRNA degradation and translational repression. In this study, microarray data from HK-2 cells exposed to CdCl₂ was analyzed, revealing increased oxidative stress and disrupted mitochondrial function. The prolonged cadmium exposure disrupted gene expression and induced persistent toxicity. Notably, six miRNAs predominantly modulated the hub genes. A molecular interaction study of miRNA-mRNA duplexes indicated a strong interaction with the argonaute (AGO) protein of the RNA-induced silencing complex (RISC), suggesting that miRNA-mediated gene silencing plays a crucial role in cadmium-induced renal damage. These findings highlight the critical role of miRNAs in modulating cadmium toxicity and suggest their potential as biomarkers for cadmium-induced renal dysfunction.

**Supplementary Information:**

The online version contains supplementary material available at 10.1038/s41598-025-11473-1.

## Introduction

CdCl₂ is a crystalline white substance widely used in industrial applications such as photocopying, dyeing, and electroplating^1,2^. Among cadmium compounds, CdCl₂ is notably hazardous, with well-documented carcinogenicity, and poses significant health risks, supported by substantial evidence from animal studies, though direct evidence in humans is limited^3^. Exposure to CdCl₂ can occur via inhalation or ingestion, leading to symptoms such as coughing, breathing difficulties, skin irritation, gastrointestinal distress, and vomiting^4^. The precise mechanisms underlying cadmium toxicity remain incompletely understood; however, prolonged treatment with chelating agents, such as quinamic acid, can mitigate some toxic effects^5^.

Environmental cadmium levels have surged due to human activities. The extensive use of cadmium-containing fertilizers and micronutrients facilitates cadmium uptake by plants, resulting in bioaccumulation in humans upon consumption^6^. Cigarette smoke is another significant source of cadmium exposure, markedly elevating blood cadmium concentrations^7^. Additionally, industrial processes, including mining, contribute to the heightened bioavailability of cadmium in human populations^8^.

Renal toxicity is a prominent consequence of cadmium exposure, with the kidney’s proximal tubular cells being particularly vulnerable^9,10^. The HK-2 cell line, derived from the proximal tubular cells of an adult male human kidney, serves as a model for studying cadmium-induced nephrotoxicity^11^. The proximal tubule, extending from the renal pole of Bowman’s capsule to the commencement of the Henle loop, is crucial for reabsorbing water, salts, and other solutes such as glucose, amino acids, phosphate, and citrate^12,13^. It also plays essential endocrine roles, including vitamin D synthesis^14^. Proximal tubular cells are implicated in the pathogenesis of various renal diseases, including Fanconi syndrome, renal tubular acidosis, Dent disease, cystinuria, and acute kidney injury^13,15^.

miRNAs are short, non-coding RNAs that regulate post-transcriptional gene expression by binding complementarily to target mRNAs. The biogenesis of miRNAs begins with RNA polymerase II transcribing miRNA genes into primary miRNAs^16^. In the nucleus, the RNase endonuclease Drosha cleaves primary miRNAs into precursor miRNAs, which are subsequently exported to the cytoplasm by Exportin 5^17^. In the cytoplasm, dicer further processes these precursors into double-stranded RNA, with one strand incorporated into the AGO-containing RISC, leading to mRNA silencing via cleavage and degradation^18,19^.

The role of miRNAs in renal toxicity and cadmium-induced nephrotoxicity is an emerging area of research^20^. miRNAs are critical regulators of gene expression and have been shown to modulate various biological processes, including apoptosis, proliferation, and differentiation, which are relevant to kidney function and injury^21,22^. Moreover, in a study by Lemaire et al., 2020, hsa-miR-146b-5p and hsa-miR-21-5p were upregulated and influenced the expression of genes linked to oxidative stress, inflammation, and cellular repair pathways in renal proximal tubular epithelial cells^20^.

Despite extensive research efforts, the precise molecular mechanisms underlying CdCl₂-induced nephrotoxicity are not yet fully elucidated. There is a pressing need for more detailed studies on the interactions between cadmium-induced oxidative stress and miRNA regulation, as well as the specific miRNA-mRNA interactions involved in renal toxicity. To address these gaps, this study aimed to investigate the regulatory role of miRNAs in cadmium-induced renal toxicity. Microarray data were utilized to profile gene expression in proximal tubular cells exposed to CdCl₂. Through the identification of differentially expressed genes and subsequent regulatory network analysis, associations with specific miRNAs were elucidated. Additionally, duplex structures were constructed with target hub genes and their associated miRNAs. Molecular interaction studies assessed the interactions between the mRNA-miRNA duplex and the AGO protein within the RISC, providing insights into the mechanisms by which miRNAs may modulate gene expression in response to cadmium exposure.

In summary, this study aims to identify miRNAs associated with CdCl₂-induced renal toxicity, contributing to the identification of biomarkers and therapeutic targets to mitigate the adverse effects of cadmium exposure on renal health. This investigation highlights the critical role of miRNAs in modulating gene expression and cellular responses under cadmium stress, thereby providing valuable insights into the molecular and structural basis of CdCl₂-induced renal damage and potential intervention strategies.

## Methodology

### Differential gene expression

The Gene Expression Omnibus (GEO) database was used to retrieve an expression profiling by array dataset (GSE27211) of time-dependent (12 and 48 h) CdCl₂ exposure of HK-2 cells in triplicates and a control against each. The microarray used a GPL570 [HG-U133_Plus_2] Affymetrix Human Genome U133 Plus 2.0 Array platform. The Differentially Expressed Genes (DEGs) were determined using the limma (v3.50.3) Bioconductor package for R (v4.1.2). DEGs were determined based on *P* value < 0.05 and log2 fold change (log2FC) > 1, with false discovery rate control using the Benjamini & Hochberg method^23^. Dimensionality reduction of the gene expression data was carried out using Uniform Manifold Approximation and Projection (UMAP) and Principal Component Analysis (PCA), implemented through the umap, FactoMineR and factoextra packages to generate visual plots. Additionally, heatmap and volcano plots were produced using the pheatmap and ggplot2 packages, respectively^24^.

### Protein-protein interaction network construction and analysis

The PPI network of DE genes was generated using the STRING database. The interaction score was set to a medium confidence level, i.e., ≥ 0.40, and FDR stringency was set to 5%. The obtained network was exported to Cytoscape for visualization and analysis. The Molecular Complex Detection (MCODE) and CytoHubba applications were used to determine the densely interconnected regions of the network^25^.

### Gene ontology and functional enrichment analysis

ShinyGo (v0.77) was utilized to identify Gene Ontology (GO) terms associated with the hub genes, explicitly focusing on biological processes and molecular functions relevant to *Homo sapiens*. A false discovery rate (FDR) threshold of < 0.05 was applied to ensure statistical significance. The FDR correction helped reduce the likelihood of false positives in GO term identification, thereby increasing the reliability and robustness of the results^26^.

### mi-RNA prediction and regulatory network construction

The associated miRNAs of the hub genes were predicted using TargetScan and miRTarBase 2022^27,28^. The miRNAs were filtered through an intersectional analysis a regulatory network was constructed using Cytoscape with hub genes as target nodes and miRNAs as source nodes. This network depicts miRNA-based regulation of the hub genes in specific biological processes.

### Duplex structure prediction of miRNA-mRNA complexes

The miRNA sequence and gene binding location were retrieved via the miRTarBase. The complexes’ secondary structure was predicted using RNAfold. This server also predicted the minimum free energy plain diagram. RNAComposer was used to build the tertiary structure of the duplex^29^. The RISC component AGO protein was retrieved (PDB 3F73). Dock Prep of Chimera was used to prepare the structure of AGO for docking- water molecules were removed, hydrogens and charges were added, and modified residues were replaced by standard residues^30^.

### Molecular interaction of miRNA duplex and argonaute

Molecular docking between the prepared AGO protein and the duplex structures was performed using PatchDock. The complex with the highest score and lowest atomic contact energy (ACE) was selected. Amino acids participating in hydrogen bonding and other non-bond interactions with the duplex were analyzed and visualized using BIOVIA Discovery Studio 3.5^29,31^.

### Molecular dynamics simulations

The MD simulations were performed on the *MRPS10*-hsa-miR-146b-5p-AGO complex, with AGO in its apo form used as a control. The system was solvated in a dodecahedral box with the TIP3P water model and neutralized by adding Na^+^ and Cl^-^ ions. The general AMBER force field (GAFF) was employed to describe the interactions within the system^32^. MD simulations were performed using GROMACS v2021.4-2. Energy minimization was carried out using 10,000 steps of the steepest descent algorithm, followed by the conjugate gradient method. Following, a 100 ps NVT equilibration was executed at 310 K using the Berendsen thermostat for solvent stabilization, followed by a 1 ns NPT equilibration to 1 atm using the Berendsen barostat. The production was conducted in the NPT ensemble with a time step of 2 fs, utilizing the v-rescale thermostat and the Parrinello-Rahman barostat. Long-range electrostatic interactions were handled via the Particle-Mesh Ewald (PME) method, while short-range electrostatic and van der Waals interactions were calculated with a 10 Å cut-off. Bond constraints were applied using the LINCS algorithm^33^.

## Results

### Differentially expressed genes

**Fig. 1:**
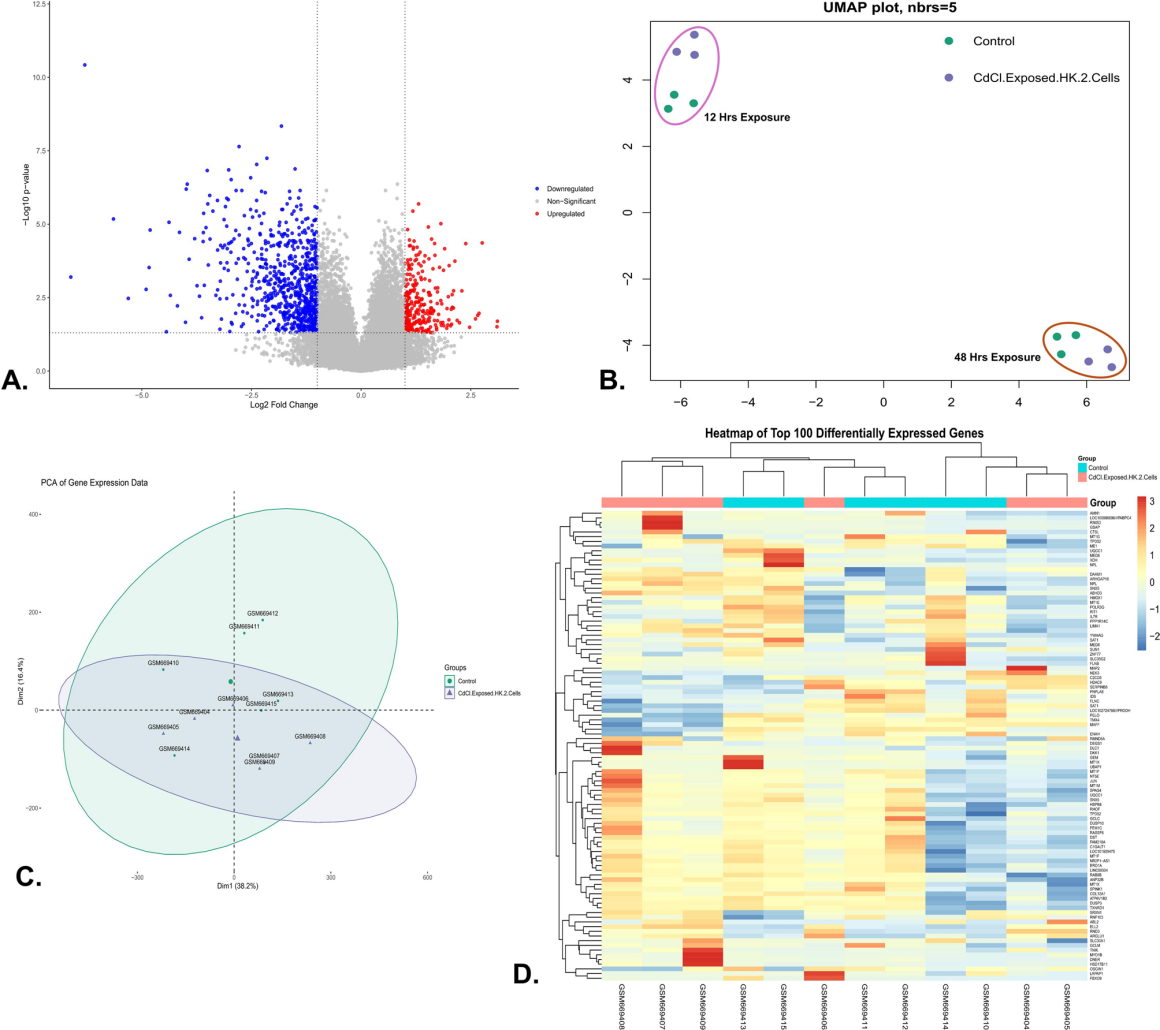
**(A)** Volcano Plot illustrating the significant DEGs with log2FC > 1 upregulated (red) and log2FC < −1 downregulated (blue) and adjusted p-value < 0.05, (**B)** The distinct clustering patterns of the HK-2 cells exposed to CdCl₂ and their controls for 12- and 48, (**C)** PCA plot of individual samples, and (**D)** Heatmap of the top 100 DEGs in the control and CdCl_2_ exposed HK-2 cells.

Based on the transcriptome data of three samples of 12-hrs and 48-hrs CdCl₂ exposed HK-2 cells and controls against each, 54,676 genes were obtained after data processing and cleaning, among which a total of 992 differentially expressed genes (DEGs) were identified (p-value < 0.05, fold change > 1), including 316 upregulated genes and 676 downregulated genes. The volcano plot (Fig. 1A) represents the significant genes that satisfy the cut-off criteria to visualize the DEGs (Fig. 1A). The UMAP (Uniform Manifold Approximation and Projection) algorithm was applied to reduce the dimensionality of non-coding RNA expression data, revealing distinct clustering patterns linked to CdCl₂ exposure time points and control conditions. The n_neighbors parameter was set to 5 to balance local and global dataset organization. The UMAP plot showed a clear separation between HK-2 cells exposed to CdCl₂ for 12 and 48 h, indicating a temporal progression in transcriptional changes (Fig. 1B). Data quality was shown by a PCA plot (Fig. 1C), and the heatmap of the top 100 DEGs is presented in Fig. 1D.

### PPI network and topology analysis

**Fig. 2:**
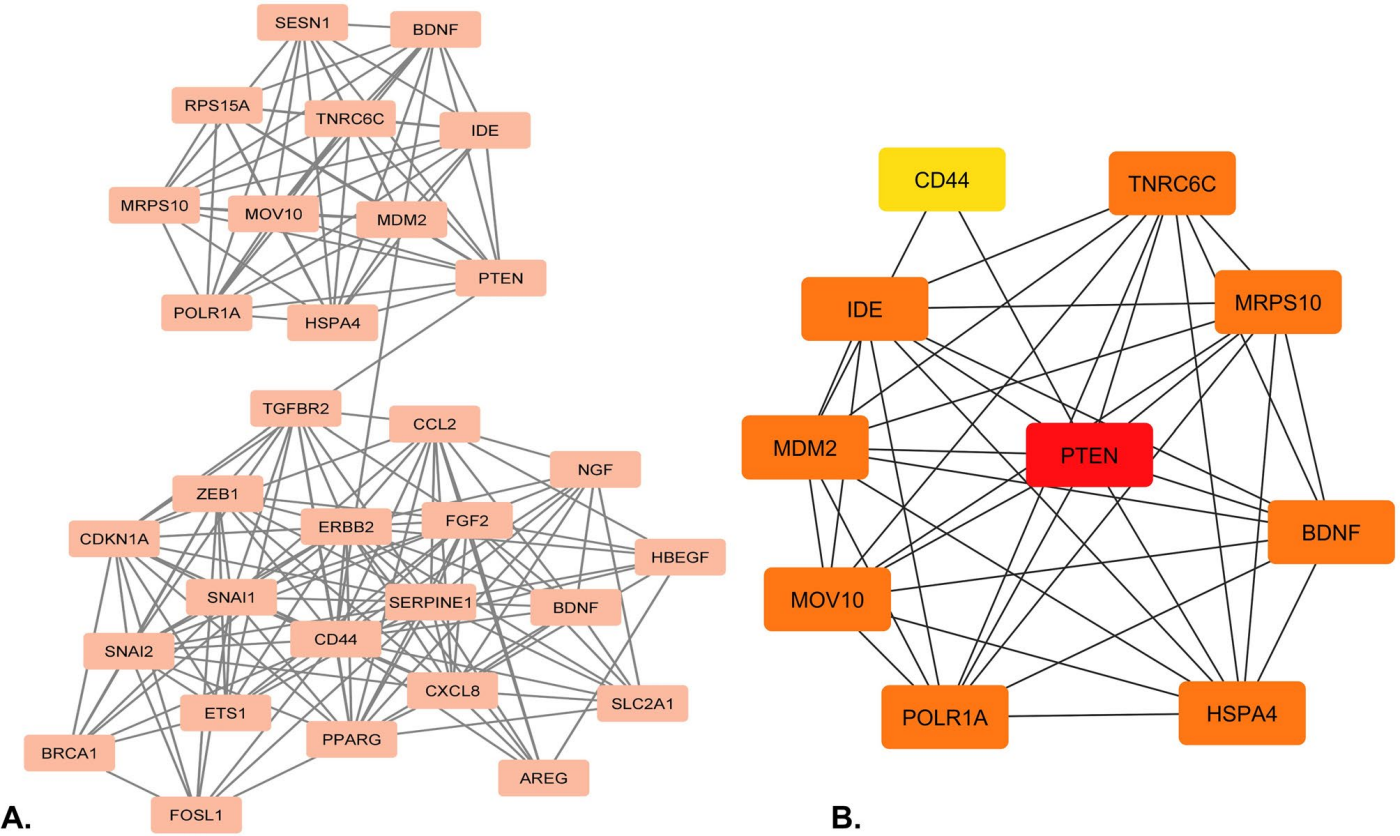
**(A)** The densely interconnected region of the PPI network and (**B)** The top ten Hub genes were determined using the MCC algorithm.

The PPI network of DE genes was generated with a medium interaction score and FDR stringency of 5%, possessing a clustering coefficient of 0.26. MCODE determined the densely interconnected region in the network with the highest cluster score of 11.88 (Fig. 2A). Further, the MCC algorithm was used to unveil hub genes since it has a greater accuracy quality in evaluating the topological relevance of nodes and is ranked based on their shortest path length. The top 10 hub genes were obtained, illustrated in Fig. 2B.

### Hub gene functional enrichment

**Fig. 3:**
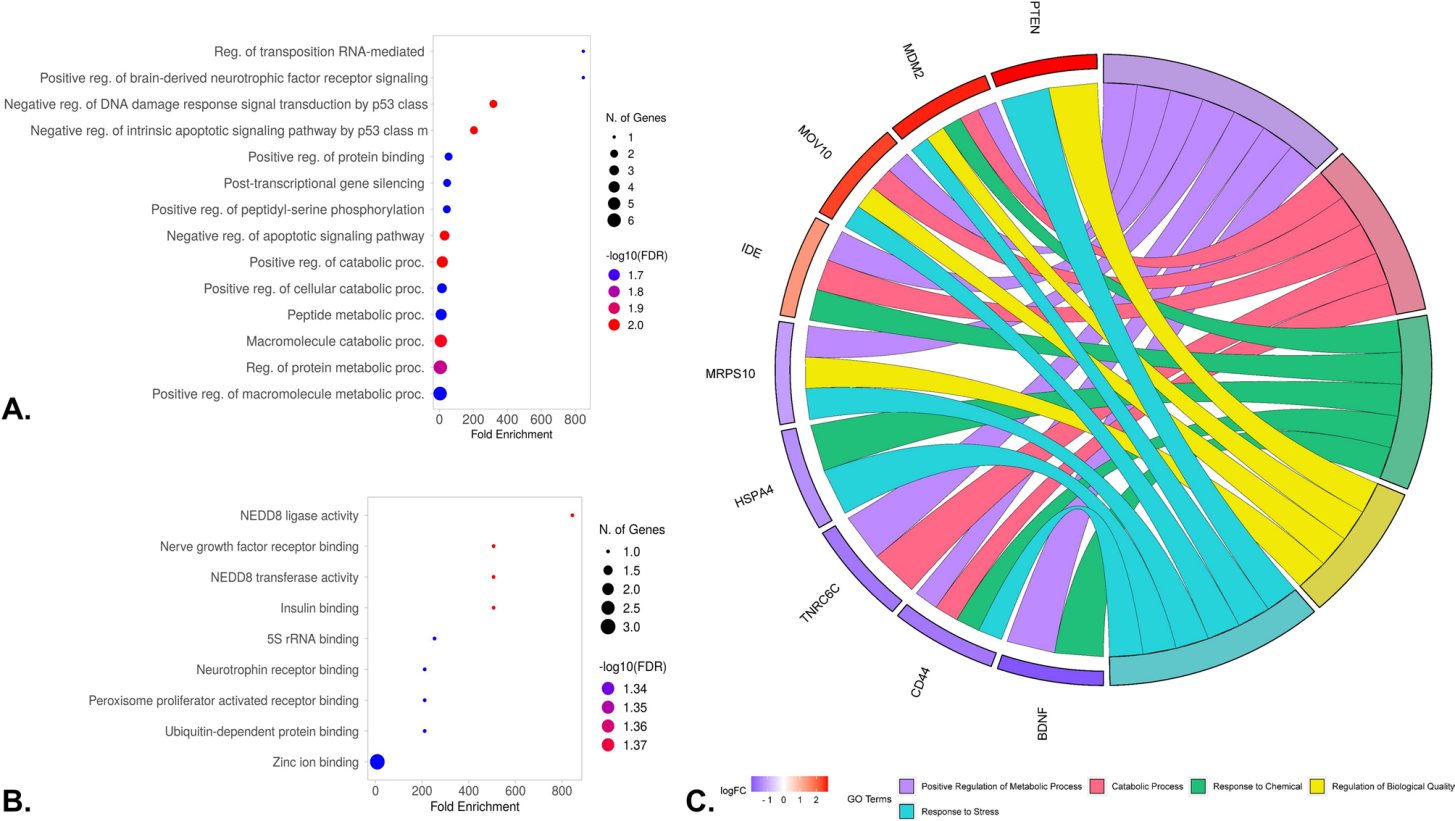
**(A)** GO terms and biological processes of the hub genes are based on fold enrichment at 5% significance. (**B)** Molecular functions associated with the hub genes. (**C)** Top 5 GO terms enriched to the hub genes and their expression pattern.

The GO and functional enrichment analysis of hub genes revealed significant involvement in various biological processes (Fig. 3A). The hub genes were predominantly enriched in biological processes related to metabolism, including positive regulation of macromolecule metabolic processes, peptide metabolic processes, protein metabolic processes, and cellular catabolic processes. Notably, the hub genes were also significantly enriched in processes related to negative regulation of apoptotic signaling pathways, particularly those mediated by *p53*. Further analysis revealed that the hub genes play important roles in cellular defense mechanisms, particularly in response to external stimuli. On the molecular function level, enrichments such as *NEDD8* ligase activity, ubiquitin-dependent protein binding, insulin binding, and zinc ion binding emphasize their involvement in post-translational modifications, protein degradation, and metabolic regulation, while peroxisome proliferator-activated receptor binding points to roles in lipid metabolism (Fig. 3B). Together, these enrichments demonstrate the multifunctional nature of the hub genes, contributing to cellular metabolism, stress response, and survival, reflecting their critical importance in maintaining cellular integrity (Fig. 3C).

### Gene regulatory network

**Fig. 4:**
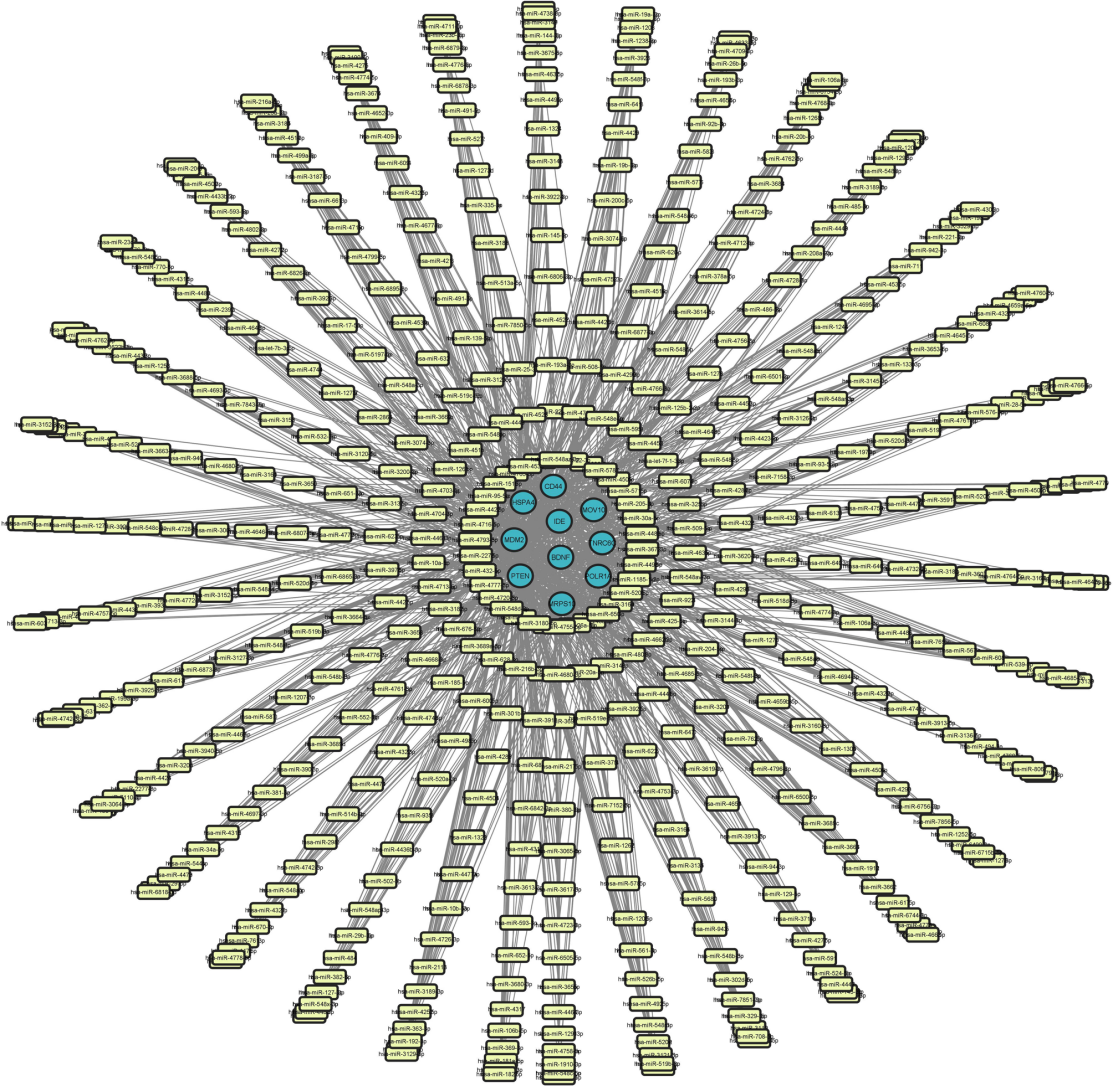
miRNA-mRNA gene regulatory network, illustrating the miRNAs modulating the expression of the target hub genes.

The related miRNAs for the hub genes were predicted using the TargetScan database and validated using mirTarbase, which offers information on empirically validated miRNA-mRNA interactions (**Supplementary File**). In contrast to both databases, overlapping miRNAs were filtered for related hub genes. Cytoscape was used to create a network of miRNA-mRNA interactions (Fig. 4). The network was assessed as a directed graph with the NetworkAnalyzer tool. The network, which has 264 nodes and 257 edges, indicates that many miRNAs modulate the hub genes in the network, which is illustrated with a regulatory network. The miRNAs hsa-miR-4257, hsa-miR-4311, and hsa-miR-4698 were discovered to have maximum association with *MRPS10*. The miRNAs, hsa-miR-146b-5p, hsa- miR-21-5p, and hsa-miR-324-5p also target the gene *MRPS10* and have additional evidence of being downregulated when HK-2 cells are exposed to CdCl₂. Due to the above-stated reasons, the miRNAs hsa-miR-146b-5p, hsa-miR-21-5p, hsa-miR-324-5p, hsa-miR-4257, hsa-miR-4311 and hsa-miR-4698 were employed for molecular interaction studies.

### mRNA-miRNA duplexes

**Fig. 5:**
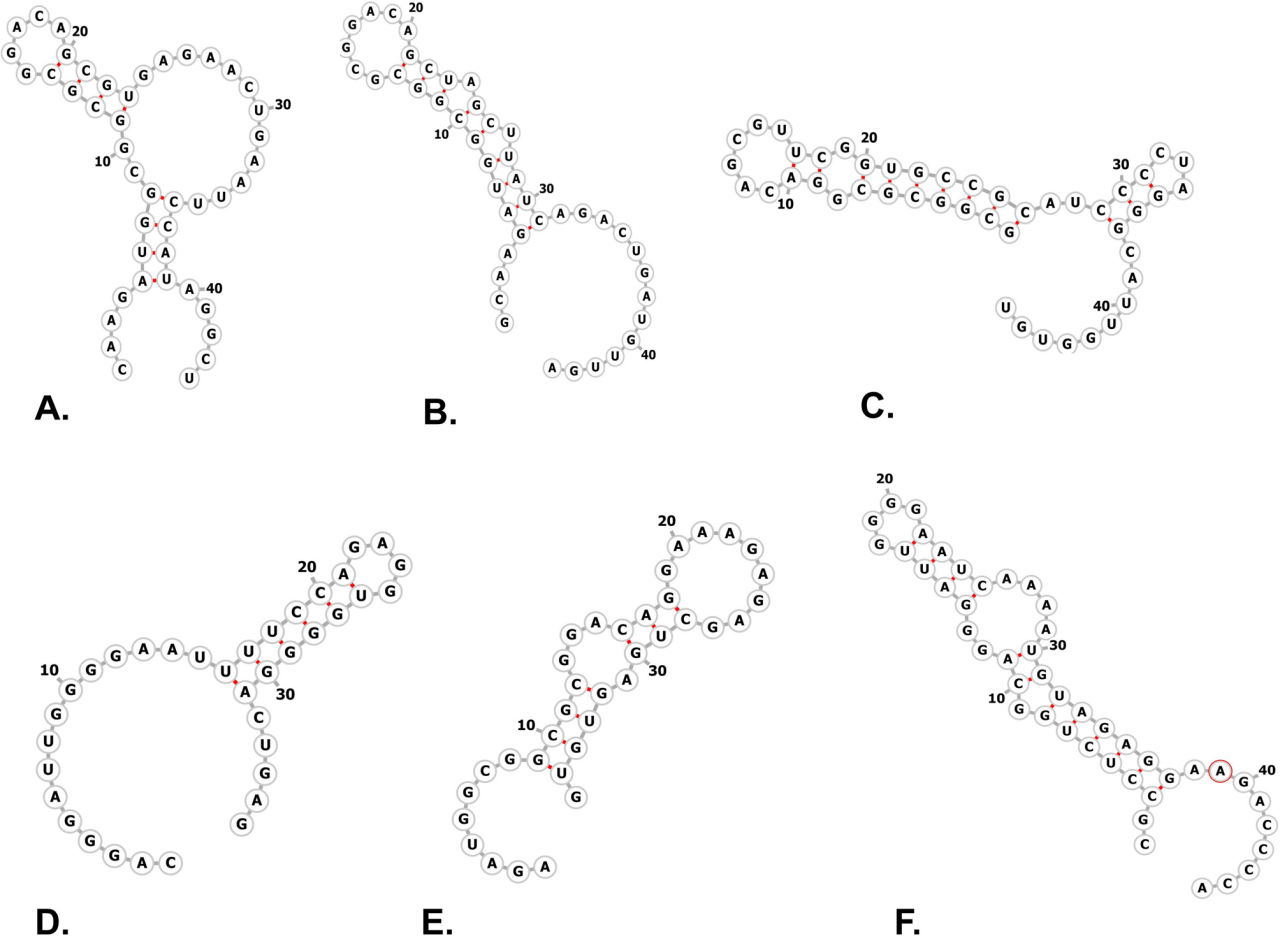
Secondary structures of miRNA-*MRPS10* duplexes. (**A)** hsa-miR-146b-5p (**B)** hsa-miR-21-5p (**C)** hsa-miR-324-5p (**D)** hsa-miR-4257 (E.) hsa-miR-4311 (**F)** hsa-miR-4698.

Six duplex structures were constructed using *MRPS10*, and the following miRNAs, hsa-miR-146b-5p, hsa-miR-21-5p, hsa-miR-324-5p, hsa-miR-4257, hsa-miR-4311 and hsa- miR-4698. The complexes’ secondary structure was predicted using the RNAfold web server, and its secondary structures are shown in Fig. 5, and binding energy values are in Table 1. The minimum free energy suggests that the duplex structures are stable. The duplex structures- *MRPS10*-hsa-miR-324-5p and *MRPS10*-hsa-miR-4698 are predicted to have high binding affinities due to their low minimum binding energies. The tertiary structures of the duplex structures were generated using RNAComposer.

**Table 1:** The secondary structure of the *MRPS10* and MiRNA duplexes. The table includes dot-bracket notation of the secondary structure and their minimum free energy in kcal/mol.

miRNA	Dot-Bracket	Minimum Free Energy (kcal/mol)
hsa-miR-146b-5p	….((((.((((….))))…………))))….	−6.4
hsa-miR-21-5p	….(((((((((…….))).)).))))………….	−7.1
hsa-miR-324-5p	(((((((.((……)).))))))).(((…)))………	−18.4
hsa-miR-4257	……………((((((….))))))….	−4.4
hsa-miR-4311	…….((((…(((………))).)))).	−4.4
hsa-miR-4698	.((((((.((.((((….))))….))))))))………	−11.2

### Molecular docking

**Fig. 6:**
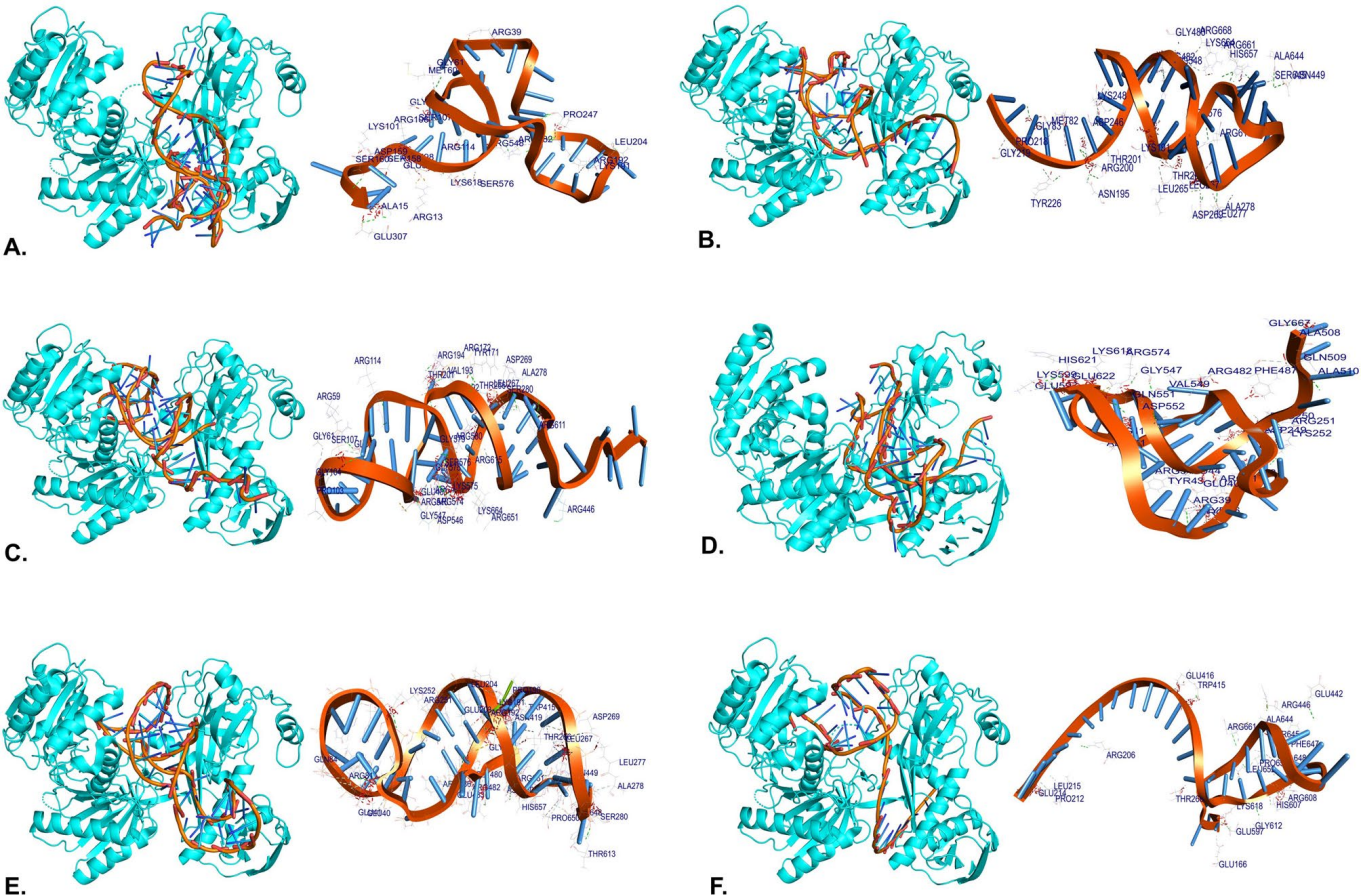
Amino acid residues of AGO protein are involved in the interactions between the *MRPS10*-miRNA duplex structures within 3.5 Å. (**A)** hsa-miR-146b-5p, (**B)** hsa- miR-21-5p (**C)** hsa-miR-324-5p, (**D)** hsa-miR-4257, (**E)** hsa-miR-4311, and (**F)** hsa-miR- 4698.

Docking was performed between the AGO protein and miRNA-mRNA duplexes using the PatchDock server. The docking score and atomic contact energies are listed in Table 2. The nature of the molecular interactions between the duplexes and the AGO protein was performed to determine the specificity of the binding of the duplexes. All amino acids interacting with the duplex within 3.5 Å were considered (Table 3**and** Fig. 6). The hydrogen bonds obtained within 2 Å were considered, which implied a strong binding affinity (Table 4).

**Table 2:** The binding affinity of the duplexes and AGO protein. The table includes the score, area, and ACE.

mRNA-miRNA Duplex and AGO	Score	Area	ACE
hsa-miR-146b-5p	25,546	3402.8	−140.72
hsa-miR-4311	19,920	3438.6	−413.40
hsa-miR-4698	18,304	4054.7	−648.61
hsa-miR-21-5p	19,154	4153.8	−249.97
hsa-miR-324-5p	19,066	3789.4	−223.88
hsa-miR-4257	18,248	3173.1	−435.31

**Table 3:** Amino acid residues of AGO protein are involved in the interactions between the duplex structures within 3.5 Å.

MRPS10-miRNA Duplex	Amino Acid Residues		
	Hydrogen Bonds	Aromatic Rings	Hydrophobic Interactions
hsa-miR-146b-5p	ARG106, ARG114, ARG13, ARG192, ARG39, ARG482, ARG548, LEU597, ARG574, ARG59, ARG192, ASP159, ASP598, GLN551, GLU307, GLU40, GLY104, GLY77, GLY61, LEU204, LYS101, LYS191, LYS618, MET60, PRO247, SER107, SER158, SER160, SER484, SER576, and ALA15	-	-
hsa-miR-21-5p	ALA508, ARG114, ARG251, ARG39, ARG482, ARG482, ARG574, ARG59, ARG81, ASP154, ASP249, ASP552, ASP552, GLN509, GLN551, GLU483, GLU597, GLU622, GLY547, GLY667, LYS248, MET82, PHE487, PRO36, and PRO37	PHE 487 and TYR 86	-
hsa-miR-324-5p	ALA278, ALA644, ALA648, ARG482, ARG192, ARG482, ARG548, ARG661, ARG668, ARG81, ASN449, ASP660, GLN84, GLU41, LEU203, GLU40, GLU483, GLY480, GLY481, GLY667, LEU204, LEU267, LEU277, LYS191, LYS191, PHE647, PRO650, THR266, and TRP415	TRP415 and PHE647	-
hsa-miR-4257	ALA644, ALA648, ARG206, ARG446, ARG608, ARG615, ARG661, GLU166, GLU214, GLU416, GLU442, GLU597, GLY612, HIS607, LEU153, LEU215, LEU652, LYS618, PHE647, PRO212, PRO650, SER645, THR266, and TRP415	PHE267 and TRP415	-
hsa-miR-4311	ALA278, ALA644, ARG200, ARG251, ARG482, ARG548, ARG615, ARG661, ARG668, ASN195, ASN449, ASP246, ASP269, GLY219, GLY480, GLY83, HIS657, LEU267, LEU277, LEU265, LYS191, LYS248, LYS664, MET82, PHE649, PRO218, SER576, SER645, THR201, THR266, and TYR226	TYR226, TRP415, and PHE649	TRP415
hsa-miR-4698	ALA278, ARG114, ARG172, ARG192, ARG194, ARG446, ARG482, ARG548, ARG574, ARG580, ARG59, ARG611, ARG615, ARG651, ASN195, ASP269, ASP546, GLU40, GLU483, GLY104, GLY547, GLY578, GLY579, GLY61, LEU267, LEU277, LYS575, LYS664, PRO103, SER107, SER280, SER576, THR201, THR266, TYR171, and VAL193	TYR171	-

**Table 4:** List of amino acid residues of AGO and atoms of the duplexes participating in hydrogen bonding within 2 Å.

miRNA-MRPS10	Residues	Atom	Distance (A°)
hsa-miR-146b-5p	ALA15	HO2’ (U44)	1.79806
	ASP159	H3’OD2 (G41)	1.8055
	ASP598	H2’OD2 (A40)	1.8128
	ARG192	HH12O5’ (G11)	1.88534
	ARG13	HH21OP1 (U39)	1.90456
	LYS618	HZ1OP2 (U39)	1.93459
	PRO247	H22O (G24)	1.96044
	ARG482	HH11O2 (C12)	1.9679
hsa-miR-21-5p	ASP249	H21OD2 (G5)	1.48255
	ARG574	HEOP1 (C13)	1.60637
	ARG482	HEN3 (A24)	1.68445
	ALA508	HAO3’ (G1)	1.73173
	LYS248	HZ1OP1 (U7)	1.73628
	GLU483	H3’OE1 (G25)	1.83035
	ASP552	H4’OD2 (G11)	1.88762
hsa-miR-324-5p	GLU41	H6OE1 (C38)	1.59723
	LEU204	HOP1 (G22)	1.60929
	GLY480	H4’O (C2)	1.70084
	ARG482	HD3OP2 (U44)	1.7083
	LYS191	HE3O5’ (U21)	1.84613
	LEU277	H5’O (A10)	1.92637
	LYS191	HZ2OP2 (G22)	1.93527
	ARG482	HH22N3 (G43)	1.94214
hsa-miR-4257	GLU416	HAOP1 (G12)	1.60874
	ARG661	HD3OP1 (G30)	1.70979
	TRP415	HOP1 (A13)	1.92354
	ARG446	HEOP1 (G27)	1.93687
hsa-miR-4311	LYS191	HE3O5’ (G32)	1.31114
	LYS191	HZ2OP2 (U33)	1.74554
	ARG482	HAOP2 (C12)	1.80388
	GLY480	HA3OP1 (C12)	1.8133
	ASP269	HO2’OD1 (A17)	1.89859
hsa-miR-4698	GLY547	H4’O (G13)	1.40664
	LEU277	H62O (A38)	1.43911
	LYS575	HZ1O4’ (U30)	1.87878
	ARG194	HH11OP2 (G9)	1.88319
	ARG482	HD3N3 (A11)	1.93114
	ARG548	HH12O2’ (U30)	1.95116

### Trajectory assessment of the complex

**Fig. 7:**
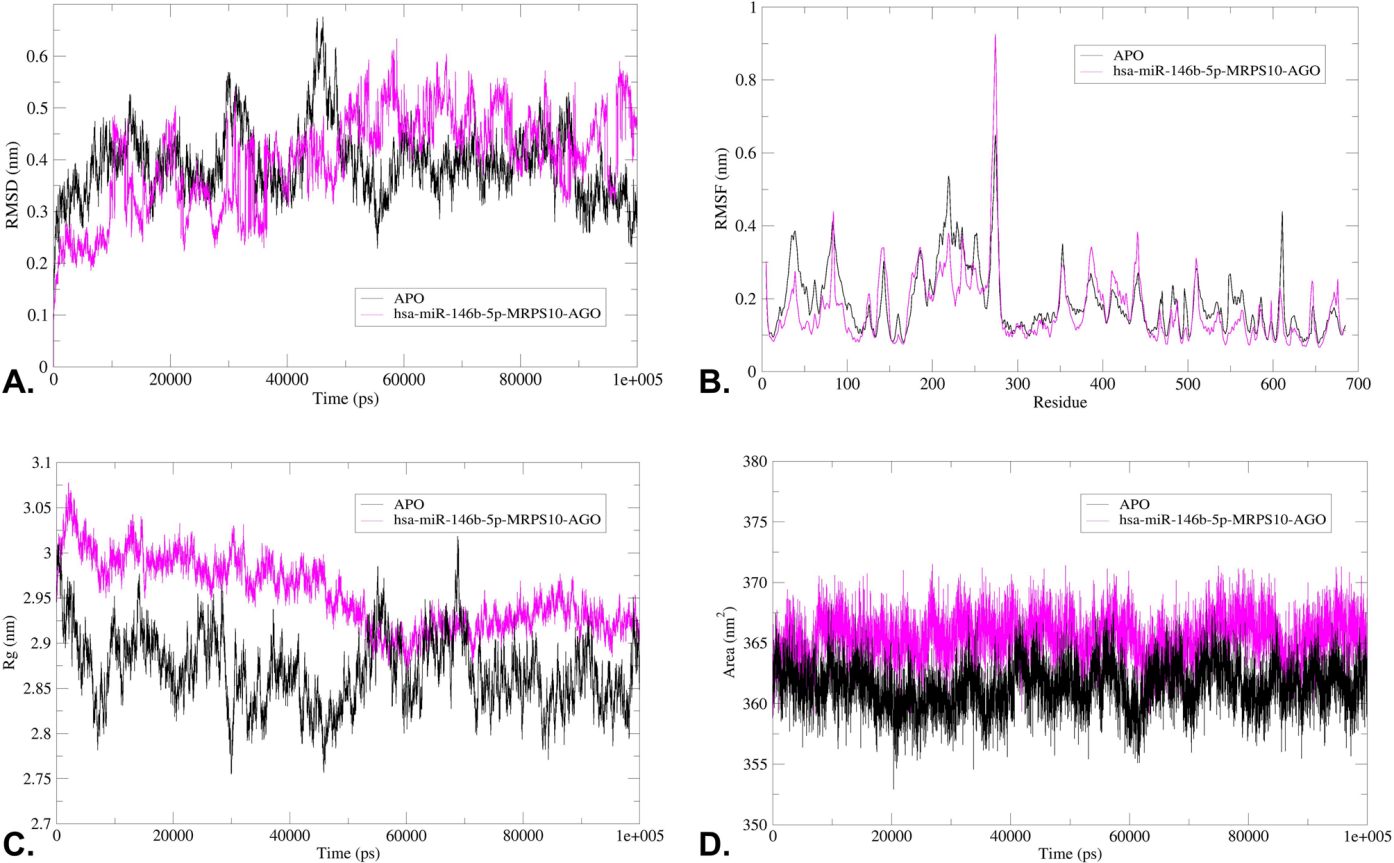
The trajectory assessment of hsa-miR-146b-5p-*MRPS10* duplex and AGO complex during a 100 ns trajectory. (**A)** RMSD, (**B)** RMSF, (**C)** Rg, and (**D)** SASA.

**Fig. 8:**
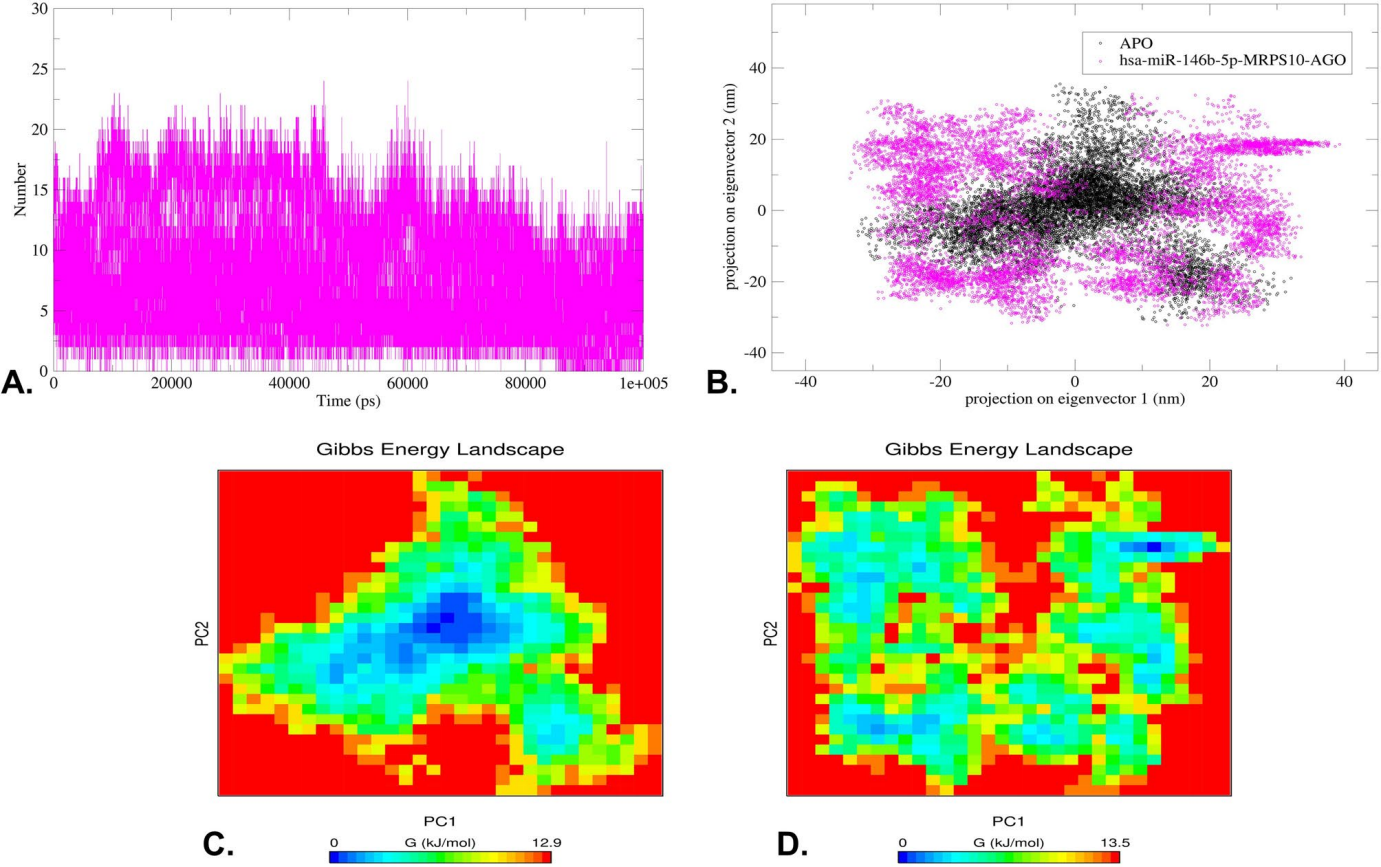
**(A)** The H-bond contacts between the duplex with AGO during 100 ns simulation, (**B)** PCA plot illustrating the 2D projections of the complexes, and (**C)** The Gibbs FEL of the APO and (**D)** hsa-miR-146b-5p-*MRPS10* duplex bound to AGO.

**Fig. 9:**
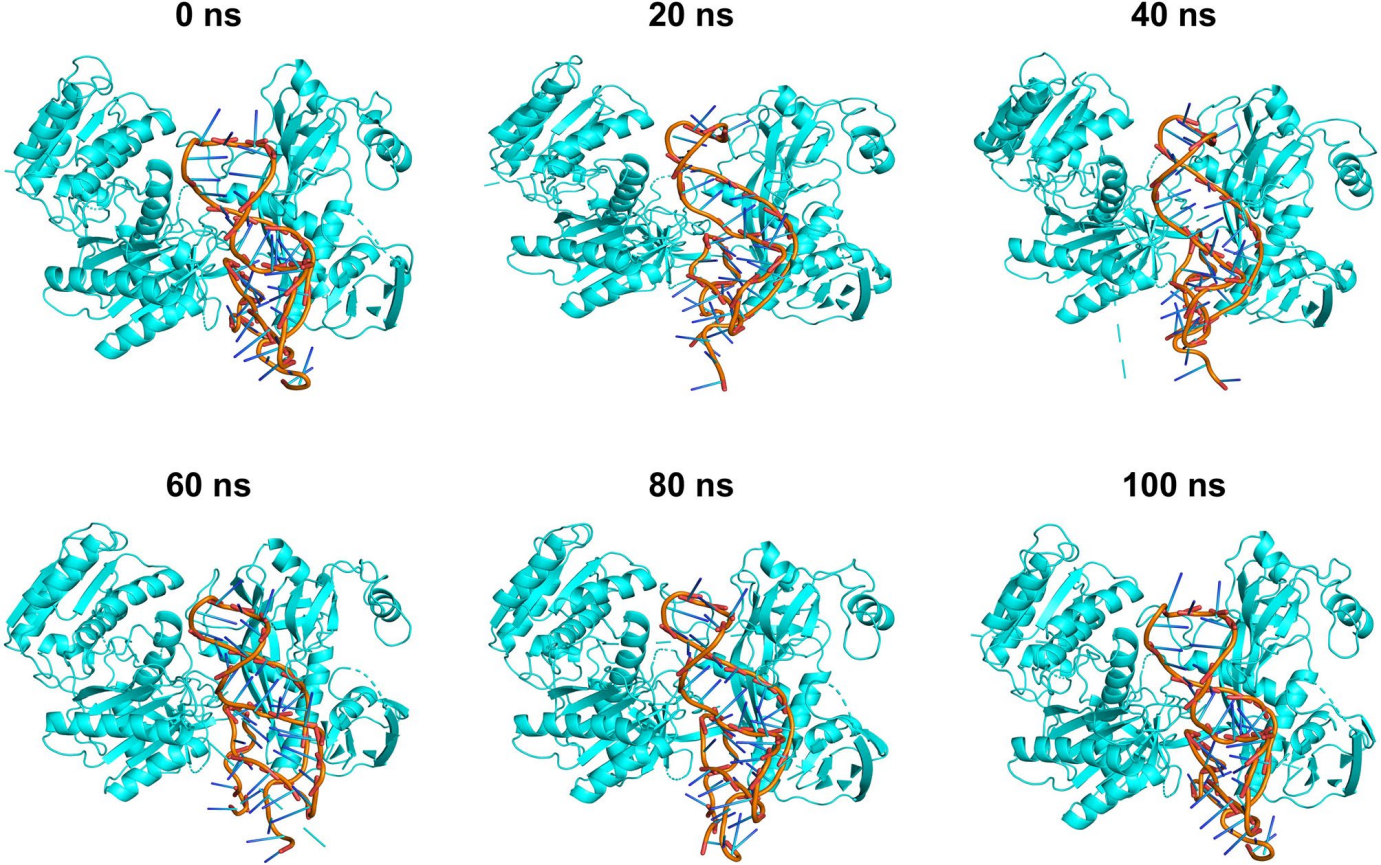
Trajectory snapshots of the complex at every 20 ns.

The analysis of the RMSD plot indicated that the binding of hsa-miR-146b-5p-*MRPS10* duplex to the AGO resulted in comparable structural deviations from its native state in contrast to APO, as depicted in Fig. 7A. The complex’s flexibility and rigidity, as elucidated through the root mean square fluctuations (RMSF) profiles of the residues within the protein backbone and interacting amino acids, reveal residual fluctuations in several regions, as depicted in Fig. 7B. The radius of gyration (Rg) suggests the uniform distribution of the atoms of the macromolecules around the axes of the protein backbone compared to APO, indicating that the complex is compact throughout the trajectory **(**Fig. 7C**)**. The accessibility of the protein’s interaction surface to the solvent reveals the conformational changes the protein underwent upon hsa-miR-146b-5p-*MRPS10* duplex binding **(**Fig. 7D**)**. The average values for these parameters are illustrated in Table 5. The number of hydrogen bonds was evaluated throughout the simulation trajectory **(**Fig. 8A**)**. H-bond interactions between the hsa-miR-146b-5p-*MRPS10* duplex and AGO were consistent throughout the trajectory as observed at 20 ns intervals, and the highest of 24 H-bonds was observed **(**Fig. 9**)**.

The PCA aided in identifying the overall motion patterns of the complex. These principle components (PCs) captured the dominant modes of motion within both the APO and the complex. The analysis of eigenvectors obtained from PCA allowed us to identify the most significant collective movements and structural changes that occurred during the simulation. Notably, the first two PCs of the APO and the complex explained the highest variance in the data. Furthermore, the 2D projection of trajectories onto the eigenvectors revealed considerable overlap between the apoprotein and the complex **(**Fig. 8B**)**.

The Gibbs FEL was constructed using 2D projections involving the first two eigenvectors, as exemplified in Fig. 8C and **D**. In this representation, regions exhibiting a deeper blue hue represent regions of lower energy. Notably, a significant observation was the complete transformation of the primary free energy well upon the binding of the hsa-miR-146b-5p-*MRPS10* duplex. The duplex binding induced a distinctive global energy minimum during the 100 ns trajectory. Moreover, the energy landscape of the complex unveiled multiple distinct minima, each corresponding to multiple metastable structural states of the complex with respect to the APO, separated by relatively modest energy barriers, which ranged from 12.9 to 13.5 KJ/mol.

**Fig. 10:**
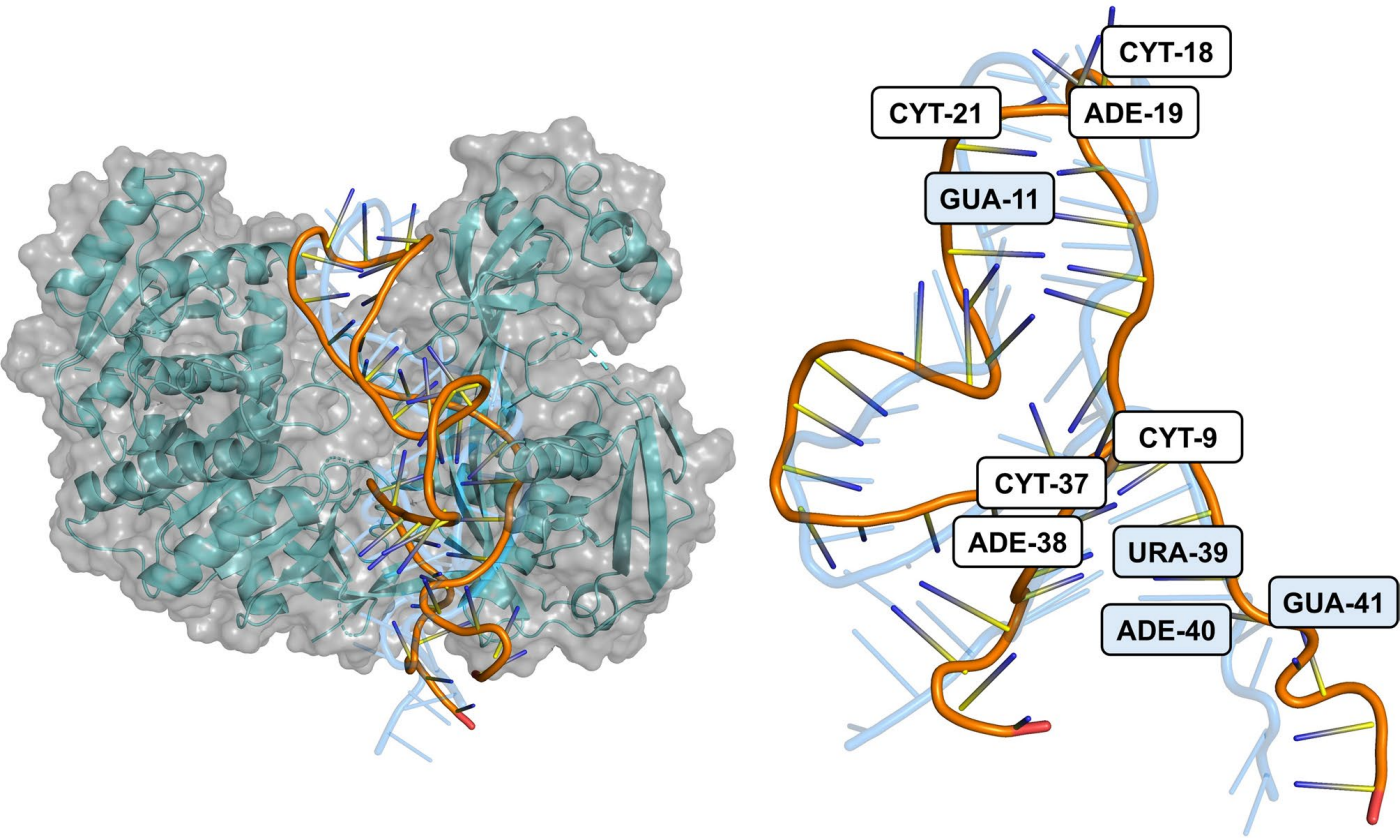
Superimposition of the duplex at 100 ns with its initial conformation bound to AGO, highlighting the nucleotides involved in hydrogen bond formation with the protein at distances < 2 Å. Four nucleotides consistently participated in hydrogen bond interactions throughout the trajectory.

The structural analysis of the hsa-miR-146b-5p-*MRPS10* duplex at 100 ns simulation trajectory, when superimposed with its initial conformation, reveals significant conformational changes upon interaction with the AGO protein within the RISC (Fig. 10). The superimposed structures indicate an expanded duplex configuration characterized by an increased number of hydrogen bonds. Notably, the 3’ terminal of the miRNA exhibited a discernible displacement away from the AGO complex, suggesting dynamic adjustments in the binding interface. This displacement reflects the inherent conformational flexibility required for optimal positioning and interaction with *MRPS10*^33^. Furthermore, a detailed analysis of the hydrogen bonding patterns reveals a higher frequency of interactions with bond lengths less than 2 Å. Specifically, the residues G11, U39, A40, and G41 of the duplex consistently formed hydrogen bonds with the AGO protein throughout the simulation trajectory. This observation demonstrates the enhanced stability and the potential for the increased binding affinity of hsa-miR-146b-5p-*MRPS10* duplex with AGO. The higher occurrence of such short hydrogen bonds is indicative of a more tightly coordinated and stable interaction network. The structural adaptations observed, including the noted folding of the 5’ terminal and the displacement of the 3’ terminal, likely facilitate the accommodation of the miRNA-mRNA duplex into the AGO protein. These conformational changes enhance the structural integrity and specificity of the miRNA-AGO complex, which are critical for the precise recognition and subsequent silencing of target mRNAs.

**Table 5:** The average RMSD, RMSF, rg, and SASA values of the complex for a 100 Ns trajectory.

Complex	RMSD (nm)	RMSF (nm)	Rg (nm)	SASA (nm^2^)
*MRPS10*-hsa-miR-146b-5p duplex	0.398	0.170	2.953	365.455
APO	0.392	0.190	2.869	361.487

## Discussion

Cadmium is abundant in the environment due to human actions such as using fossil fuels, igniting metal ore, and waste incineration. The transport of cadmium compounds to the plants from sewage sludge seeping into agricultural soil may play a critical role in the food chain and accumulate throughout many human organs^5^. Cadmium is a key industrial component and pollutant that contributes significantly to diseases associated with the kidney. The consensus is that cadmium accumulates in proximal tubule cells; it causes a broad range of adverse effects that end up with the death of renal epithelial cells via necrotic or apoptotic pathways^9^. The primary site for the cadmium deposition is the proximal tubules due to the enlargement of its epithelial cells, resulting in polyuria and proteinuria^34^. Cadmium exposure in humans is predicted to impair miRNA expression, resulting in gene expression dysregulation and harmful consequences. However, research on miRNA regulation in response to CdCl₂ exposure remains sparse, particularly in proximal tubular cells.

We further explored the current dataset and determined the change in gene expressions based on log2FC among the CdCl₂-exposed and normal HK-2 cells. The PPI network was used to understand the physical and functional association between the significant DEGs. A distinct cluster was identified, characterized by highly interconnected regions within the network, providing deeper insights into key genes, their interactions, and their roles in regulating biological processes. The GO and Functional enrichment analysis reveals a strong association between these hub genes and critical biological processes. The enrichment of hub genes in these metabolic pathways suggests that both anabolic and catabolic activities are affected during cadmium toxicity, thereby influencing the balance between cellular growth, repair, and energy production^35^. Moreover, the positive regulation of protein binding and post-transcriptional gene silencing indicate that cadmium toxicity may impact protein stability and gene expression control at multiple levels^36,37^. Disruption of these processes under cadmium stress could lead to aberrant protein synthesis and contribute to cellular dysfunction^38^.

One of the notable findings is the negative regulation of apoptotic signaling pathways, specifically those mediated by the tumor suppressors. The *p53* pathway is well known for its role in regulating apoptosis in response to DNA damage and stress, including cadmium-induced oxidative damage^39^. The downregulation of apoptotic pathways under cadmium exposure suggests that cells might engage in survival mechanisms to evade apoptosis, thereby promoting cellular repair and adaptation to toxic insults^40^. However, prolonged exposure to cadmium may eventually overwhelm these defense mechanisms, leading to cell death^41^.

The response to chemical and response to stress processes further highlights the role of these genes in cellular defense mechanisms. Cells exposed to cadmium activate stress response pathways to counteract the toxic effects of the metal, including the induction of antioxidant enzymes and detoxification pathways^41,42^. The upregulation of *PTEN* (Phosphatase and tensin homolog) in this context is particularly significant. *PTEN* is a well-established regulator of cell survival and proliferation, acting through the inhibition of the PI3K/AKT pathway^43^. Its upregulation may serve as a protective response against cadmium-induced stress, limiting cell proliferation and promoting DNA repair^44^.

In contrast, the downregulation of *MRPS10* (Mitochondrial Ribosomal Protein S10) could impair mitochondrial protein synthesis and disrupt energy production, contributing to mitochondrial dysfunction, a hallmark of cadmium toxicity^41,45^. This suggests that cadmium may exert its toxic effects in part through disruption of mitochondrial function, leading to energy depletion and oxidative stress. Furthermore, the disruption of *MRPS10* altered the energy balance and enabled the injured cells to proliferate with intrinsic oxidative DNA damages by upregulating E3 ubiquitin-protein ligase Mdm2, a protein that modulates DNA damage response and signal transduction by the *p53* class mediator^46^. As a result of acute cadmium overload, ROS, which is involved in tissue destruction, is produced. Therefore, prolonged Cadmium tolerance with impaired gene expression is crucial in persistent Cadmium toxicity and carcinogenesis^47^.

On the molecular function level, several enriched GO terms underscore the complexity of cadmium toxicity. Enrichments such as *NEDD8* ligase activity, ubiquitin-dependent protein binding, insulin binding, and zinc ion binding point to the importance of post-translational modifications and protein degradation in response to cadmium exposure^48,49^. For instance, *NEDD8*, a ubiquitin-like protein, plays a crucial role in the regulation of protein degradation through the ubiquitin-proteasome system^46^. Disruption of these pathways can lead to impaired cellular function. The involvement of zinc ion binding is particularly relevant, given the role of zinc in maintaining the structural integrity of many proteins, including transcription factors and enzymes^50,51^. Cadmium, being a chemical analog of zinc, can displace zinc from these proteins, leading to their inactivation and contributing to cadmium’s toxicity. This molecular mimicry may explain some of the disruptions observed in zinc-binding proteins and highlight the importance of metal homeostasis in cellular survival under cadmium stress. Additionally, the enrichment in peroxisome proliferator-activated receptor (PPAR) binding suggests a role in lipid metabolism. PPARs are nuclear receptors that regulate lipid metabolism and energy homeostasis^52^. Their binding by cadmium-responsive hub genes indicates that lipid metabolic pathways were dysregulated, contributing to cellular dysfunction and energy imbalance.

The importance of miRNAs in crucial physiological processes, such as oxidative stress, inflammation, and apoptosis, and their ability to respond to metal toxicity has piqued our interest and could serve as critical molecular switches for adjusting cellular stress responses. Protein-coding genes and other non-coding transcripts are both negatively regulated by them. These miRNAs have a sequence-specific influence on gene expression, notably in gene silencing through the assembly of the RISC complex, which includes the argonaute protein. The complementary RISC complex activates the AGO protein’s endonuclease activity, targeting mRNA breakage, and alters the association between AGO and the 3′ ends of the miRNA, aiding its degradation^16,53^.

A network pharmacology approach was used to determine potential miRNAs linked to the hub genes^54^initiated with mRNA complementarity to the seed region of miRNAs, and continued to the 23 bases, demonstrating valid relationships^55^. The relationships are then evaluated using a metric system, experimental evidence, and text mining to improve the discovery of literature relating to miRNA-mRNA interactions^56^. A miRNA-mRNA interaction network reveals that different miRNAs target many mRNAs in the module, which uncovered that *MRPS10*, inducing apoptosis tolerance in the HK-2 cells, was regulated by six overlapping miRNAs, hsa-miR-146b-5p, hsa-miR-21-5p, hsa-miR-324-5p, hsa-miR-4257, hsa-miR-4311, and hsa-miR-4698). The minimal free energies of these miRNAs for *MRPS10* imply that the binding relationship was energetically favorable, as demonstrated by the binding affinity of the duplexes and the prediction of the optimum secondary structures^57^. The molecular interactions between putative miRNAs-*MRPS10* duplex that bind to the argonaute protein in the RISC complex were studied.

Molecular docking was employed to understand the molecular interactions between the miRNA-AGO and duplexes-AGO protein. Furthermore, the docking technique included molecule shape complementarity and scoring. While evaluating each feasible transformation, a scoring method considered geometric fit and atomic desolvation energy^58^. The higher the score, the lower the atomic contact energy. The docking uncovered the interactions involving hydrophobic interactions, aromatic rings between the amino acid residues of the protein, and specific atoms in the duplexes within 3.5Å, along with H bond interactions. H bonding and pi stacking were the most prominent interactions and were vital for gene regulation^31^. Aliphatic amino acids with high hydrophobicity, such as isoleucine, leucine, valine, and alanine, increase stability during the interaction^29^. The molecular docking analysis indicated that the protein’s amino acid residues were involved in hydrogen bond interactions with different atoms of duplexes within 2Å. H bond interactions stabilize the conformations and promote strong binding and selectivity^59,60^.

The MD simulation analysis was employed to investigate the behavior of the complex in dynamic states and assess the stability of the *MRPS10*-hsa-miR-146b-5p duplex with AGO over a 100 ns duration. The significance of this study lies in its comprehensive evaluation of the dynamic interactions and stability of these complexes, which are crucial for understanding mRNA degradation potential. The low and stable RMSD values observed for the complex, in contrast to the APO, suggest stable interactions, with the RMSD fluctuations attaining convergence after specific time points in the trajectory, indicating equilibration^61^. This stability is further corroborated by the RMSF analysis, which identified regions of higher stability at the active sites of the proteins, aligning with the molecular docking results^62^.

The Rg values suggest a more condensed backbone, which could be attributed to modifications in protein-duplex interactions^63^. The SASA analysis further revealed that miRNA duplex binding comparable SASA values to APO, indicating tighter protein folding and stability^64^.

Hydrogen bond analysis, a crucial determinant of complex stability, showed varying numbers of hydrogen bonds throughout the simulation, maintaining consistent hydrogen bonding^65^. This variability suggests that some complexes exhibit high and stable interactions^66^. PCA illustrated increased collective motion of the complex compared to the APO, indicating greater flexibility or larger conformational changes upon duplex binding^67^. The FEL further depicted the conformational changes, with multiple distinct minima corresponding to metastable states, highlighting the dynamic nature of the complexes^68^. These changes may enhance the protein’s ability to mediate mRNA degradation through RISC. The duplex interacted with AGO primarily through hydrogen bonding and hydrophobic interactions, leading to significant conformational changes that affected the protein’s structure^69^. These interactions occurred at specific binding sites on the protein, where the polar side chains of amino acids facilitated hydrogen bond formation^70^. The observed structural changes and increased hydrogen bonding reflect the adaptive flexibility and stability of the *MRPS10*-hsa-miR-146b-5p duplex within the AGO complex. These features are essential for the effective functioning of the RISC in gene silencing mechanisms, providing insights into the molecular basis of miRNA-mediated gene regulation of *MRPS10*^31^.

In summary, this study uncovered the miRNA-based negative regulation of the hub genes involved in impaired regulation of DNA damage response, signal transduction by the *p53*, and cellular protein metabolic process. Potential miRNAs modulated the target-specific genes in HK-2 cells when exposed to CdCl₂. The Molecular interaction analysis provided a detailed understanding of the dynamic interactions of *MRPS10*-hsa-miR-146b-5p duplex-bound to AGO in the RISC complex. However, this study has certain limitations. It does not comprehensively capture context-specific miRNA activity, which may vary depending on miRNA expression levels, stability, and cellular stress conditions upon CdCl₂ exposure. While the molecular modelling offered high-resolution insights into the dynamic behaviour and structural stability of complexes but do not capture the complexity of intracellular localization. Integrating the epitranscriptomic information and dose-time resolved perturbation datasets along with targeted in vitro and in vivo validation will strengthen mechanistic understanding and translational relevance.

## Conclusions

This study elucidates the molecular mechanisms underlying CdCl₂ toxicity in proximal tubular cells by focusing on miRNA-mediated gene regulation. Through network pharmacology and molecular dynamics simulations, we identified key hub genes, which were predominantly regulated by hsa-miR-146b-5p, hsa-miR-21-5p, hsa-miR-324-5p, hsa-miR-4257, hsa-miR-4311, and hsa-miR-4698. These miRNAs played a crucial role in impairing the DNA damage response and signal transduction pathways, including the *p53*-mediated apoptotic signaling pathway, thereby promoting apoptotic tolerance and cell proliferation despite intrinsic oxidative DNA damage. The findings also demonstrate the disruption of mitochondrial functions, altered energy balance, and ROS regulation, which are central to cadmium toxicity. The molecular docking and dynamics simulations provided insights into the stability and interactions between hsa-miR-146b-5p -*MRPS10* duplex and the AGO protein within the RISC complex, further highlighting the role of hsa-miR-146b-5p in modulating gene expression in response to cadmium stress. These findings provide a foundation for the strategic development of miRNA-targeted therapies to mitigate cadmium toxicity in both in vitro and in vivo settings.

## Electronic supplementary material

Below is the link to the electronic supplementary material.

Supplementary Material 1

## Data Availability

The datasets used or analyzed during the current study are available from the GEO database (https://www.ncbi.nlm.nih.gov/geo/query/acc.cgi? acc=GSE27211).
